# **Three new species of** ***Macvicaria*** **Gibson & Bray, 1982 (Digenea: Opecoelidae) infecting** ***Diplodus*** **spp. (Teleostei, Sparidae) from South Africa and Namibia**

**DOI:** 10.1007/s11230-026-10275-x

**Published:** 2026-05-27

**Authors:** Anja Vermaak, Olena Kudlai, Russell Q-Y. Yong, Nico J. Smit

**Affiliations:** 1 Water Research Group, Unit for Environmental Sciences and Management, North-West University, Potchefstroom Campus, Private Bag X6001, Potchefstroom, 2520 South Africa; 2 State Scientific Research Institute Nature Research Centre, Akademijos, 2, 08412 Vilnius, Lithuania

## Abstract

To date, only a single species of the digenean genus *Macvicaria* Gibson & Bray, 1982—*Macvicaria obovata* (Molin, 1859)—has been reported from southern African coastal waters, infecting three fish species (two sparids and one cheilodactylid). Given the general paucity of information on digeneans from marine fishes in this region, the present study aimed to explore the diversity of these parasites in the endemic sparids the Cape white seabream *Diplodus capensis* (Smith) and the zebra seabream *Diplodus hottentotus* (Smith). By integrating molecular data (based on 28S rDNA, ITS2 rDNA, *cox*1 mtDNA and *nad*1 mtDNA) with morphometric and morphological characterisation, three new species of *Macvicaria* were identified from these fish hosts in South Africa and Namibia. These are described herein as *Macvicaria umbungu*, *Macvicaria tsitsikamma* and *Macvicaria peetvermaaki*. This study represents the first record of species of *Macvicaria* from *D*. *capensis* and is the first report of a representative of this genus from Namibia. The newly generated molecular data will be valuable for future phylogenetic studies, accurate species identification, and linking different life stages of *Macvicaria* spp. in southern Africa. This study provides insight into the endemism of marine fish trematodes in southern Africa and explores biogeographical links between southern Africa and the Mediterranean fish trematode fauna.

**Supplementary Information:**

The online version contains supplementary material available at 10.1007/s11230-026-10275-x.

## Introduction

The Opecoelidae Ozaki, 1925 is the largest digenean family with numerous subfamilies, genera, and species that occur in freshwater and marine teleost fishes (Cribb, 2005; Bray et al., 2016; Martin et al., 2020a). With 18 reported species, the Opecoelidae represents the second most commonly reported marine trematode family from southern Africa (Gavrilyuk-Tkachuk, 1979; Bray, 1986, 1987; Parukhin, 1989; Vermaak et al., 2023a; Dumbo et al., 2025). Most of the opecoelid genera known from this region are represented by a single species, with the exceptions of *Coitocaecum* Nicoll, 1915 (4 species), *Helicometra* Odhner, 1902 (3 species) and *Opecoelina* Manter, 1934 (2 species) (Bray, 1987; Parukhin, 1989; Vermaak, 2018, 2020; Vermaak et al., 2023a). Given the richness of opecoelids globally, the recorded richness of this trematode family from southern Africa is relatively low, a fact attributable to historically limited sampling. Considering the high rate of regional fish endemism, the true richness of opecoelids in the region is likely much higher than currently recognised.

One genus that has received less attention in southern Africa is *Macvicaria* Gibson & Bray, 1982. This genus was proposed by Gibson and Bray (1982) to accommodate species of *Plagioporus* Stafford, 1904 (*sensu lato*) parasitising marine fishes. Many of these taxa have long and complicated taxonomic histories that have yet to be satisfactorily resolved through molecular phylogenetic analyses or thorough morphological re-assessment of type material. Currently, *Macvicaria* includes 55 recognised species (WoRMS, 2025), with most reported from the Mediterranean, Antarctica, and Australia. These species infect a wide range of teleost fishes across 42 families. The seabream and porgy family (Sparidae) is well represented as hosts, with 13 species of *Macvicaria* infecting sparids—some infecting sparids exclusively, and others parasitising both sparids and hosts from other families. Knowledge on the southern African fauna of *Macvicaria* is limited to two species, namely *Macvicaria obovata* (Molin, 1859), which was reported from Gqeberha (formerly Port Elizabeth) on the south coast of South Africa (Bray, 1987), and *Macvicaria selachophidii* Reimer, 1987, which was described from off Mozambique (Reimer, 1987). *Macvicaria obovata* [originally described as *Distomum obovatum* (Molin, 1859) from the gilthead seabream *Sparus aurata* (L.) from off Italy] was recorded as *Pachycreadium obovata* (Molin, 1859) from the zebra seabream *Diplodus cervinus* (Lowe) and musselcracker seabream *Sparodon durbanensis* (Castelnau) (both sparids), as well as the redfingers *Cheilodactylus fasciatus* Lacépède (Cheilodactylidae) (Bray, 1987); whereas *M*. *selachophidii* was described from the barbed brotula *Selachophidium guentheri* Gilchrist (Ophidiidae) (Reimer, 1987). There have been no records of *Macvicaria* spp. from Namibia.

The taxonomy of the genus *Macvicaria* remains uncertain due to the highly conserved morphology between putative species, with several species [e.g. *M. obovata* and *Macvicaria crassigula* (Linton, 1910)] likely representing complexes of genetically distinct, but morphologically cryptic, lineages (Antar et al., 2015). Recent molecular studies focusing on elucidating the taxonomic complexity of the Opecoelidae have also demonstrated the polyphyletic status of *Macvicaria*, with species of this genus forming clades spread across three opecoelid subfamilies (Bray et al., 2016; Faltýnková et al., 2017; Martin et al., 2020a). The unresolved systematic position and classification of various taxa ascribed to the genus *Macvicaria* highlights the need for further studies to refine its composition and clarify its phylogenetic position within the family.

During comprehensive investigations of the parasite diversity of the Cape white seabream *Diplodus capensis* (Smith) and the zebra *Diplodus hottentotus* (Smith) in southern Africa, specimens of *Macvicaria* were recorded from several localities across South Africa and Namibia (Vermaak, 2024). Herein, these new species of *Macvicaria* from *D. capensis* and *D. hottentotus* in South Africa, as well as from *D. capensis* in Namibia, are characterised using morphological, morphometric and molecular data. Furthermore, this study aimed to resolve their phylogenetic relationships within the subfamily Opistholebetinae alongside other species of *Macvicaria*.

## Materials and methods

### Specimen collection

**Fig. 1:**
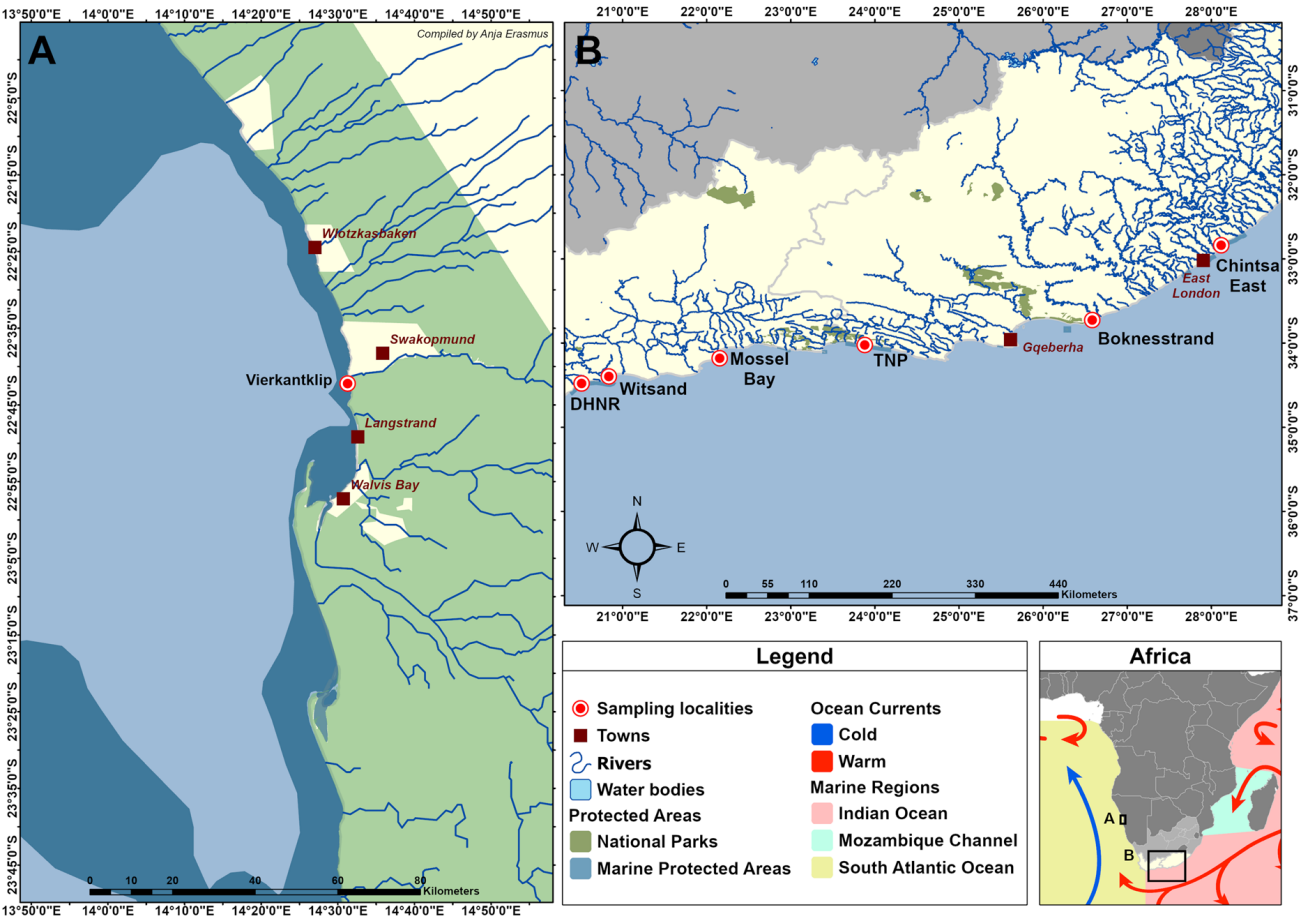
Map of sampling localities in Namibia (**A**) and South Africa (**B**). *DHNR* De Hoop Nature Reserve, *TNP* Tsitsikamma section of the Garden Route National Park

In total, 91 specimens of *D. capensis* (total length ranging between 90 mm and 360 mm) were collected from seven localities along the coast of southern Africa between 2017 and 2023: De Hoop Nature Reserve (DHNR) (n = 12), Witsand (n = 3), Mossel Bay (n = 5), the Tsitsikamma section of the Garden Route National Park (TNP) (n = 34), Chintsa East (n = 18) and Boknesstrand (n = 4), South Africa, as well as Swakopmund, Namibia (n = 15) (Fig. 1). A single *D. hottentotus* (total length 290 mm) was also collected at DHNR. Parasite collection and processing follow Vermaak et al. (2023a). Fish nomenclature follows FishBase (Froese & Pauly, 2025).

The following permits were granted for sampling: MALH-K2016-005a and SMIT-NJ/2020-004 for TNP; RES2020/29, RES2021/49 and RES2022/44 for Chintsa East, Mossel Bay and Witsand; CN44-87-18289 for De Hoop Nature Reserve; and RPIV010252022-01 for Swakopmund, Namibia.

### Morphological analyses

The selection of hologenophores and the morphological processing of specimens were done according to Vermaak et al. (2023a). Detailed drawings and measurements were made using a Nikon Eclipse 80*i* microscope with an attached drawing tube and digitised using Adobe Illustrator v. 28.5 and Adobe Photoshop v. 25.9.1. All measurements, unless otherwise stated, are in micrometres (μm) and given as ranges with means in parentheses. Voucher material, including holotypes and paratypes, were deposited in the National Museum Bloemfontein (NMB) and the Iziko South African Museum, Cape Town, South Africa (ISAM).

### Molecular analyses

#### Generation of molecular data

Following the recommendations of Blasco-Costa et al. (2016) and Cribb et al. (2025), DNA sequences of various genetic markers—including ITS1-5.8S-ITS2, ITS2, partial 18S and 28S rDNA, and the mitochondrial genes *cox*1 and *nad*1—were generated for specimens collected in the present study. DNA extractions were performed as described by Vermaak et al. (2023a). Hologenophores were made of representative specimens to generate complementary morphological vouchers and molecular sequences, following Pleijel et al. (2008). Information on the gene regions amplified by polymerase chain reaction (PCR), primers used, and PCR protocols is provided in Table 1.

**Table 1:** Primers and protocols used for the generation of new sequences

Locus	Primer name (F/R/IF/IR)	Sequence	Reference	Protocol used/reference for protocol used
28S	Digl2 (F)	5′- AAG CAT ATC ACT AAG CGG -3′	Tkach et al. (2001)	Tkach et al. (2003)
	1500R (R)	5′- GCT ATC CTG AGG GAA ACT TCG -3′	Snyder & Tkach (2001)	
	ECD2 (IR)	5′- CTT GGT CCG TGT TTC AAG ACG GG -3′	Tkach et al. (2003)	
	300F (IF)	5′- CAA GTA CCG TGA GGG AAA GTTG -3′	Littlewood et al. (2000)	
18S	18SU467F (F)	5′- ATC CAA GGA AGG CAG CAG GC -3′	Suzuki et al. 2006	Vermaak et al. (2023b)
	18SL1310R (R)	5′- CTC CAC CAA CTA AGA ACG GC -3′	Suzuki et al. 2006	
ITS1-5.8S-ITS2	D1 (F)	5′- AGG AAT TCC TGG TAA GTG CAAG -3′	Galazzo et al. (2002)	Galazzo et al. (2002)
	D2 (R)	5′- CGT TAC TGA GGG AAT CCT GGT -3′	Galazzo et al. (2002)	
	S18 (F)	5′- TAA CAG GTC TGT GAT GCC -3′	Jousson et al. (1999)	95°C for 5 min; 40 cycles: 94°C for 30 s, 53°C for 45 s, 72°C for 2 min; and 72°C for 7 min
	L3T (R)	5′- CAA CTT TCC CTC ACG GTA CTTG -3′	Jousson et al. (1999)	
	S20T2 (IF)	5′- GGT AAG TGC AAG TCA TAA GC -3′	Jousson et al. (1999)	
	L5 (IR)	5′- TTC ACT CGC CAT TACT -3′	Jousson et al. (1999)	
ITS2	3S (F)	5′- GGT ACC GGT GGA TCA CGT GGC TAG TG -3′	Morgan & Blair (1995)	Kudlai et al. (2015)
	ITS2.2 (R)	5′- CCT GGT TAG TTT CTT TTC CTC CGC -3′	Cribb et al. (1998)	
*cox*1	Dig_Cox1FA (F)	5′- ATG ATW TTY TTY TTY YTD ATG CC -3′	Wee et al. (2017)	Wee et al. (2017)
	Dig_Cox1R (R)	5′- TCN GGR TGH CCR AAR AAY CAA AA -3′	Wee et al. (2017)	
	DICE1F (F)	5′- ATT AAC CCT CAC TAA ATT WCN TTR GAT CAT AAG -3′	Van Steenkiste et al. (2015)	Vermaak et al. (2023b)
	DICE14R (R)	5′- TAA TAC GAC TCA CTA TAC CHA CMR TAA ACA TAT GATG -3′	Van Steenkiste et al. (2015)	
*nad*1	NDJ11 (F)	5′-AGA TTC GTA AGG GGC CTA ATA-3′	Kostadinova et al. (2003)	Kostadinova et al. (2003)
	NDJ2a (R)	5′-CTT CAG CCT CAG CAT AAT-3′		

*F* forward primer, *IF* internal forward primer, *IR* internal reverse primer, *R* reverse primer

The resultant PCR products were visualised with 1% agarose gel electrophoresis and sent to a commercial sequencing company for purification and sequencing (Inqaba Biotechnical Industries (Pty). Ltd., Pretoria, South Africa). Novel sequences were assembled and edited using Geneious Prime v. 2025.2.1 bioinformatics software (Biomatters, Auckland, New Zealand) and were deposited in GenBank (Table 2).

**Table 2:** Summary data for the sequences of *Macvicaria* spp. from *Diplodus capensis* and *Diplodus hottentotus* generated in the present study

Host	Locality	GenBank accession numbers					
		18S	ITS2	28S	*cox*1	H	*nad*1
***Macvicaria peetvermaaki***							
*D*.* capensis*	Swakopmund, Namibia	–	PZ068336	PZ068325	PZ067593	H1	–
		–	–	–	PZ067594	H1	–
		–	–	–	PZ067595	H3	–
		–	–	–	PZ067596	H4	–
		–	–	–	PZ067597	H2	–
***Macvicaria tsitsikamma***							
*D*.* capensis*	TNP, SA	–	PZ068337	PZ068326	PZ067598	H1	PZ117060
		–	PZ068338	PZ068327	PZ067599	H2	
***Macvicaria umbungu***							
*D. capensis*	TNP, SA	–	PZ068339		PZ067600	H1	
		–	–	–	PZ067601	H7	PZ117061
		–	–	–	PZ067602	H7	PZ117062
	DHNR, SA	–	PZ068340	PZ068328	PZ067603	H4	PZ117063
		–	PZ068334*	PZ068329	PZ067604	H8	PZ117064
		–	–	–	PZ067605	H11	–
		–	–	–	PZ067606	H1	PZ117065
		–	–	–	PZ067607	H1	PZ117066
	Mossel Bay, SA	–	PZ068341	PZ068330	PZ067608	H1	PZ117067
		–	PZ068342	PZ068331	PZ067609	H5	PZ117068
		–	–	–	PZ067610	H1	PZ117069
	Boknes Bay, SA	–	–	–	PZ067611	H1	PZ117070
		–	–	–	PZ067612	H1	PZ117071
		–	–	–	PZ067613	H3	–
		–	–	–	PZ067614	H1	–
		–	–	–	PZ067615	H1	–
	Chintsa East, SA	PZ068324	–	–	PZ067618	H2	PZ117072
		–	–	–	PZ067619	H6	PZ117073
*D. hottentotus*	DHNR, SA	–	PZ068335*	PZ068332	PZ067616	H1	–
	TNP, SA	–	PZ068343	–	PZ067617	H9	–
	Chintsa East, SA	–	–	PZ068333	PZ067620	H10	–

*H* haplotype, *DHNR* De Hoop Nature Reserve, *TNP* Tsitsikamma section of the Garden Route National Park, *SA* South Africa. * Isolates with the whole ITS1-5.8S-ITS2 region amplified

### Phylogenetic analyses

Novel sequences were aligned with the sequences representing members of all currently recognised subfamilies within the family Opecoelidae (Martin et al., 2020b) retrieved from GenBank (Table 3). The outgroups for the analyses were selected based on the phylogenetic analyses in Martin et al. (2020b) and Pérez-Ponce de León & Hernández-Mena (2019). Four alignments (partial 28S rDNA, ITS2 rDNA, *cox*1 mtDNA, and *nad*1 mtDNA) were generated using MUSCLE (Edgar, 2004) implemented in Geneious v. 2025.2.1 and used for phylogenetic analyses. Nucleotide substitution models were estimated using jModelTest v. 2.1.4 (Posada, 2008). Based on the Akaike information criterion, the best model for the phylogenetic analyses of the 28S and ITS2 alignments was the general time reversible model with gamma distribution rate variation among sites and estimates of invariable sites (GTR + I + G). Phylogenetic relationships for these gene regions were estimated using Bayesian inference (BI) and maximum likelihood (ML) methods, whereas the neighbour-joining (NJ) method was used for analysis of the *cox*1 mtDNA gene. MrBayes software (ver. 3.2.3) was used to perform BI analyses, and ML analyses were performed using PhyML v. 3.0 (available at http://www.atgc-montpellier.fr/phyml/). Parameters for these analyses were set as described in Vermaak et al. (2023b), with the only modification being the number of simulated generations for the 28S and ITS2 analyses being increased from 3,000,000 to 10,000,000. Neighbour-joining analysis of Kimura-2-parameter distances was performed using MEGA v. 11 (Tamura et al., 2021); nodal support was estimated using 1,000 bootstrap replicates. The *cox*1 mtDNA alignment was aligned with reference to the amino acid translation, using the trematode mitochondrial code (translation table 21; https://www.ncbi.nlm.nih.gov/Taxonomy/Utils/wprintgc.cgi#SG21) (Garey & Wolstenholme, 1989; Ohama et al., 1990). The *cox*1 mtDNA dataset was translated and inspected in Mesquite v 3.03 (Maddison & Maddison 2020) for stop codons per Huston et al. (2021), tested for non-stationarity caused by base composition bias using the χ^2^ test function on PAUP* (Swofford, 2002), and for substitution saturation using Xia’s test function on DAMBE 7 (Xia, 2017). No base composition bias was detected on any codon positions (P = 1.00 for all codon positions), nor any substitution saturation (I_ss_ = 0.3124; I_ss.c_ = 0.6226). Genetic distance matrices were calculated in MEGA v. 11. In addition, species delimitation analysis was performed with the Bayesian version of the Poisson Tree Processes model (bPTP) (Zhang et al., 2013) using the *cox*1 mtDNA tree estimated via BI analysis. This analysis was performed using the bPTP web server (Zhang et al., 2013; available at http://species.h-its.org/ptp) with default parameters.

**Table 3:** Sequences retrieved from GenBank and used for phylogenetic analyses

Species	Host	Locality	GenBank accession numbers		Reference
			28S	ITS2	
*Bathycreadium brayi*^a^ Pérez-del-Olmo, Dallarés, Carrassón & Kostadinova, 2014	*Trachyrincus scabrus* (Rafinesque)	Barcelona, Spain	JN085948	–	Constenla et al. (2011)
*Buticulotrema termichthysi* Bray, Waeschenbach, Dyal, Littlewood & Morand, 2014	*Thermichthys hollisi* (Cohen, Rosenblatt & Moser)	South East Pacific Rise	KF733984	–	Bray et al. (2014)
*Cainocreadium labracis* (Dujardin, 1845)	*Steromphala adansonii* ^b^ (Payraudeau)	Ebro Delta, Spain	JQ694144	–	Born-Torrijos et al. (2012)
*Choerodonicola arothokoros* Martin, Cribb, Cutmore & Huston, 2018	*Scarus ghobban* Fabricius	Moreton Bay, Australia	MG844418	–	Martin et al. (2018a)
*Coitocaecum capense* Bray, 1987	*Clinus superciliosus* (L.)	Garden Route NP, SA	OR129143	–	Vermaak et al. (2023a)
*Dimerosaccus oncorhynchi* (Eguchi, 1931)	*Oncorhynchus masou* (Brevoort)	Kedrovaya River, Russia	FR870254	–	Shedko et al. (2015)
*Fairfaxia cribbi* Hassanine & Gibson, 2005	*Lethrinus atkinsoni* Seale	Ningaloo Reef, Australia	MN081755	–	Martin et al. (2020b)
*Gaevskajatrema perezi* (Mathias, 1926)	Unspecified	Near Corsica, France	AF184255	–	Tkach et al. (2001)
	*Symphodus roissali* (Risso)	Corsica, France	–	AJ241800	Jousson et al. (1999)
*Hamacreadium mutabile* Linton, 1910	*Lutjanus griseus* (L.)	Northern Gulf of Mexico	KJ001209	–	Andres et al. (2014)
*Helicometra fasciata* (Rudolphi, 1819)	*Epinephelus fasciatus* (Forsskål)	New Caledonia	KU320597	–	Bray et al. (2016)
*Helicometra manteri* Andres, Ray, Pulis, Curran & Overstreet, 2014	*Prionotus alatus* Goode & Bean	Gulf of Mexico	KJ701238	–	Andres et al. (2014)
*Heterolebes maculosus* Ozaki, 1935	*Diodon liturosus* Shaw	Lizard Island, Australia	–	MH933872	Martin et al. (2018b)
	*Diodon hystrix* L.	Australia	AY222211	–	Olson et al. (2003)
*Holsworthotrema chaoderma* Martin, Huston, Cutmore & Cribb, 2019	*Kyphosus gladius* Knudsen & Clements	Point Peron, Australia	MK052938	–	Martin et al. (2019)
*Macvicaria alacris* (Looss, 1901)	*Labrus merula* L.	Corsica, France	–	AJ241801	Jousson et al. (1999)
*Macvicaria bartolii* Antar, Georgieva, Gargouri & Kostadinova, 2015	*Diplodus annularis* (L.)	Bay of Bizerte, Tunisia	KR149464	KR149471	Antar et al. (2015)
			–	KR149472	
*Macvicaria crassigula* (Linton, 1910)	*Diplodus vulgaris* (Geoffroy Saint-Hilaire)	Bouzedjar, Algeria	MF166846	MF166837	Rima et al. (2017)
			–	MF166834	
			–	MF166835	
			–	MF166836	
		Corsica, France	–	AJ241803	Jousson et al. (1999)
	*Tricolia speciosa* (Megerle von Mühlfeld)		–	AJ241815	
	*Paracentrotus lividus* (Lamarck)		–	AJ241814	
	*Diplodus annularis*		–	AJ277372	Jousson et al. (2000)
	*Calamus bajonado* (Bloch & Schneider)	Gulf of Mexico	–	KJ701237	Andres et al. (2014)
*Macvicaria dubia* (Stossich, 1905)	*Oblada melanura* (L.)	Bay of Bizerte, Tunisia	KR149469	KR149488	Antar et al. (2015)
			–	KR149489	
*Macvicaria gibsoni* Rima, Marzoug, Pérez-del-Olmo, Kostadinova, Bouderbala & Georgieva, 2017	*Diplodus* *vulgaris*	Bouzedjar, Algeria	MF166842	MF166830	Rima et al. (2017)
			–	MF166831	
			–	MF166832	
			–	MF166833	
*Macvicaria maamouriae* Antar, Georgieva, Gargouri & Kostadinova, 2015	*Sparus aurata* L.	Bizerte Lagoon, Tunisia	KR149467	KR149473	Antar et al. (2015)
	*Lithognathus mormyrus* (L.)		–	KR149482	
	*Sparus aurata*	Bouzedjar, Algeria	–	MF166838	Rima et al. (2017)
*Macvicaria macassarensis* (Yamaguti, 1952)	*Lethrinus miniatus* (Forster)	Heron Island, Australia	AY222208	–	Olson et al. (2003)
*Macvicaria magellanica* Laskowski, Jeżewski & Zdzitowiecki, 2013	*Patagonotothen* spp.	Antarctica	KU212191	–	Hildebrand et al. (2016)
*Macvicaria maillardi* Bartoli, Bray & Gibson, 1989	*Sparus* *aurata*	Corsica, France	–	AJ277373	Jousson et al. (2000)
*Macvicaria mormyri* (Stossich, 1885)	*Sparus* *aurata*	Bouzedjar, Algeria	MF166849	MF166840	Rima et al. (2017)
	*Lithognathus* *mormyrus*	Corsica, France	–	AJ241802	Jousson et al. (1999)
*Macvicaria muraenolepidis* Zdzitowiecki, 1990	*Muraenolepis marmorata* Günther	Ross Sea, Antarctica	MH161432	–	Sokolov et al. (2018)
*Macvicaria obovata* (Molin, 1859)	*Steromphala* *adansonii* ^b^	Ebro Delta, Spain	JQ694146	JQ694149	Born-Torrijos et al. (2012)
	*Sparus* *aurata*	Corsica, France	–	AJ241816	Jousson et al. (1999)
*Macvicaria pennelli* (Leiper & Atkinson, 1914)	*Trematomus bernacchii* Boulenger	Antarctica	MH892477	–	Faltýnková et al. (2017)
*Macvicaria* sp.	*Trematomus newnesi* Boulenger	Antarctica	MH892476	–	Faltýnková et al. (2017)
*Maculifer diodontis* Martin, Huston, Cutmore & Cribb, 2019	*Diodon* *hystrix*	Heron Island, Australia	MH933879	MH933873	Martin et al. (2018b)
*Magnaosimum brooksae* Martin, Crouch, Cutmore & Cribb, 2018	*Tripodichthys angustifrons* (Hollard)	Moreton Bay, Australia	MG813907	MG813905	Martin et al. (2018e)
*Neolebouria georgiensis* Gibson, 1976	*Notothenia coriiceps* Richardson	Galindez Island, Antarctica	ON123035	–	Faltýnková et al. (2022)
*Neopycnadena tendu* (Bray & Justine, 2007)	*Pseudobalistes fuscus* (Bloch & Schneider)	New Caledonia	FJ788506	–	Bray et al. (2009)
*Opecoeloides fimbriatus* (Linton, 1934)	*Micropogonias undulatus* (L.)	Gulf of Mexico	KJ001211	–	Andres et al. (2014)
*Opistholebes amplicoelus* Nicoll, 1915	*Lagocephalus lunaris* (Bloch & Schneider)	Moreton Bay, Australia	MH933875	MH933868	Martin et al. (2018b)
*Pachycreadium carnosum* (Rudolphi, 1819)	*Dentex dentex* (L.)	Corsica, France	–	AJ241799	Jousson et al. (1999)
*Parallelolebes virilis* Martin, ribu, Cutmore & Cribb, 2018	*Meuschenia hippocrepis* (Quoy & Gaimard)	Tasmania, Australia	MH933880	MH933874	Martin et al. (2018b)
*Peracreadium idoneum* (Nicoll, 1909)	*Anarhichas lupus* L.	North Sea, United Kingdom	AY222209	–	Olson et al. (2003)
*Peracreadium characis* (Stossich, 1886)	*Diplodus puntazzo* (Walbaum)	Corsica, France	–	AJ241796	Jousson et al. (1999)
*Plagioporus loboides* ^c^ (Curran, Overstreet & Tkach, 2007)	*Fundulus nottii* (Agassiz)	Mississippi, USA	EF523477	–	Curran et al. (2007)
*Plagioporus shawi* (McIntosh, 1939)	*Oncorhynchus tshawytscha* (Walbaum)	Oregon, USA	KX553951	–	Fayton & Andres (2016)
*Podocotyle atomon* (Rudolphi, 1802)	*Littorina saxatilis* (Olivi)	White Sea, Russia	MH161437	–	Sokolov et al. (2018)
*Podocotyloides australis* Martin, Cutmore & Cribb, 2018	*Diagramma labiosum* ^d^ Macleay	Dunwich, Australia	MF805696	–	Martin et al. (2018c)
*Podocotyloides parupenei* (Manter, 1963)	*Mulloidichthys vanicolensis* (Valenciennes)	Lizard Island, Australia	MF926409	–	Martin et al. (2017b)
*Polypipapiliotrema citerovarium* Martin, Cutmore & Cribb in Martin, Sasal, Cutmore, Ward, Aeby & Cribb, 2018	*Chaetodon quadrimaculatus* Gray	Raivavae, French Polynesia	MH823957	–	Martin et al. (2018d)
*Polypipapiliotrema heniochi* Martin, Cutmore & Cribb in Martin, Sasal, Cutmore, Ward, Aeby & Cribb, 2018	*Heniochus chrysostomus* Cuvier	Mo’orea, French Polynesia	MF926406	–	Martin et al. (2018c)
*Propycnadenoides philippinensis* Fischthal & Kuntz, 1964	*Gymnocranius grandoculis* (Valenciennes)	New Caledonia	KU320604	–	Bray et al. (2016)
*Pseudoheterolebes diodontis* (Cable, 1956)	*Diodon* *hystrix*	Heron Island, Australia	MH933876	MH933869	Martin et al. (2018b)
*Pseudoheterolebes stellaglobulus* Martin, Ribu, Cutmore & Cribb, 2018	*Dicotylichthys punctulatus* Kaup	Moreton Bay, Australia	MH933877	–	Martin et al. (2018b)
*Pseudoplagioporus labiatus* Martin, Cutmore & Cribb, 2019	*Monotaxis grandoculis* (Forsskål)	Lizard Island, Australia	MN081751	–	Martin et al. (2020b)
*Pseudopycnadena fischthali* Saad-Fares & Maillard, 1986	*Diplodus* *vulgaris*	Bouzedjar, Algeria	MF166851	MF166841	Rima et al. (2017)
*Scorpidotrema longistipes* Aken’Ova & Cribb, 2003	*Scorpis georgiana* Valenciennes	Point Peron, Australia	MK052936	–	Martin et al. (2019)
*Trilobovarium parvvatis* Martine, Cutmore & Cribb, 2017	*Lethrinus nebulosus* (Forsskål)	Lizard Island, Australia	KY551562	–	Martin et al. (2017a)
**OUTGROUPS**					
*Fairfaxia cribbi* Hassanine & Gibson, 2005	*Lutjanus gibbus* (Forsskål)	Japan	–	MN078314	Martin et al. (2020b)
*Pseudoplagioporus interruptus* Durio & Manter, 1968	*Lethrinus harak* (Fabricius)	Lizard Island, Australia	–	MN078304	Martin et al. (2020b)
*Stephanostomum pristis* (Deslongchamps, 1824)	*Phycis phycis* (L.)	Corsica, France	DQ248222	–	Bray et al. (2005)
*Zalophotrema hepaticum* Stunkard & Alvey, 1929	*Zalophus californianus* (Lesson)	USA	AY222255	–	Olson et al. (2003)

^a^Reported as *Bathycreadium elongatum* in paper; ^b^Reported as *Gibbula adansonii* in paper; ^c^Reported as *Plagiocirrus loboides* in GenBank; ^d^Reported as *Diagramma pictum labiosum* in paper

The *cox*1 mtDNA alignment was used in the haplotype network analysis. Haplotypes were identified and assigned in DnaSP v. 6.12 (Rozas et al., 2017), and networks generated and visualised using the Median Joining Network function in PopART v 1.7 (Leigh & Bryant, 2015; http://popart.otago.ac.nz/).

## Results

The specimens collected from the endemic fishes *D*. *capensis* and *D*. *hottentotus* in the present study align with the concept of *Macvicaria* as defined by Gibson & Bray (1982), Bartoli et al. (1989) and Cribb (2005). Integrative analyses of both molecular and morphological data identified these specimens as belonging to three distinct species. The first of these species is widespread in South Africa from De Hoop Nature Reserve in the Western Cape to Chintsa East in the Eastern Cape. It was found in *D*. *capensis* throughout this range, whereas *D*. *hottentotus* was only collected at De Hoop Nature Reserve and Chintsa East, where it was infected. The second species was only found in *D*. *capensis* from the Tsitsikamma section of the Garden Route National Park, and the third species was only found in *D*. *capensis* from Namibia. No specimens of *Macvicaria* were found in fish examined from Witsand. All three species are distinct from all other known species of the genus and are described below as new to science.

### Morphological characterisation

Family Opecoelidae Ozaki, 1925

Subfamily Opistholebetinae Fukui, 1929

Genus *Macvicaria* Gibson & Bray, 1982

### ***Macvicaria peetvermaaki*** n. sp.

#### Taxonomic summary

Type-host: Cape white seabream, *Diplodus capensis* (Smith) (Sparidae).

Type-locality: Vierkantklip, south of Swakopmund, Namibia (22°42'11.9" S, 14°31'16.9" E).

Site of infection: Intestine.

Type-material: Holotype (NMB P1214) and 12 stained and permanently mounted paratype specimens (NMB P1215–1226), as well as 10 ethanol-preserved specimens (NMB P1194–1196) deposited in NMB; three stained and permanently mounted paratype specimens deposited in ISAM (SAMC-A100000).

Representative DNA sequences: one partial sequence of 28S rDNA (PZ068325), one sequence of ITS2 rDNA (PZ068336), and five sequences of *cox*1 mtDNA (PZ067593–PZ067597).

Zoobank ID: The species *Macvicaria peetvermaaki *was registered in ZooBank under the LSID urn:lsid:zoobank.org:act:85FCE5C0-BA03-4DA6-85DE-22D1C16D1347.

Etymology: The species is named in honour of the late Mr. Peet Vermaak, father of Anja Vermaak, in recognition of his support of the first author’s academic endeavours on fish parasites and for his role in fostering her lifelong love for nature and conservation.

**Fig. 2:**
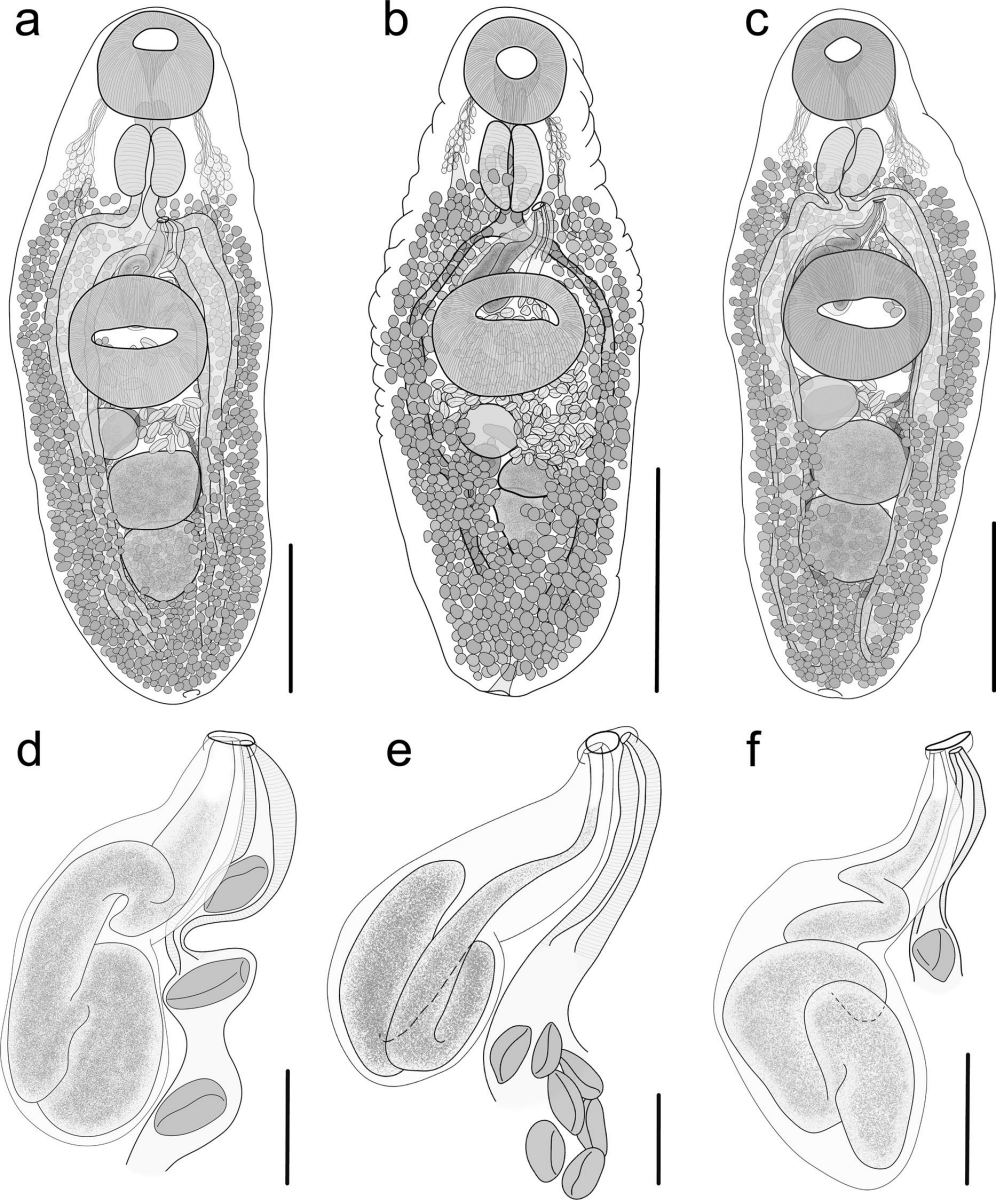
Holotypes of newly described *Macvicaria* spp., ventral view. *Macvicaria peetvermaaki* (**a**, full body; **d**, terminal genitalia), *Macvicaria tsitsikamma* (**b**, full body; **e**, terminal genitalia) and *Macvicaria umbungu* (**c**, full body; **f**, terminal genitalia). Scale bars: 500 μm (**a**, **c**); 1000 μm (**b**); 100 μm (**d**‒**f**)

Description (based on 13 whole mounts; Fig. 2a, d; Table 4, S1)

**Table 4:** Morphometric measurements of the three new species of *Macvicaria*

Species	*Macvicaria peetvermaaki*	*Macvicaria tsitsikamma*	*Macvicaria umbungu*
Host	*Diplodus capensis*	*D*. *capensis*	*D*. *capensis*, *Diplodus hottentotus*
Locality	Namibia: Swakopmund	South Africa: Tsitsikamma section of the Garden Route National Park	South Africa: De Hoop Nature Reserve, Mossel Bay, Tsitsikamma section of the Garden Route National Park, Chintsa East, Boknesstrand
Number of specimens	13	2	13
Body length	2211–3198 (2549)	1911–3008 (2460)	1867–3326 (2348)
Body width	844–1044 (939)	834–1258 (1046)	717–1297 (928)
Forebody length	764–1138 (900)	737–1119 (928)	671–1152 (826)
Hindbody length	1447–2060 (1649)	1174–1889 (928)	1167–2174 (1521)
Body width : length	1:2.43–3.06 (2.71)	1:2.29–2.39 (2.34)	1:2.25–2.78 (2.47)
Body width as % body length	32.65–41.20 (37.10)	41.82–43.64 (42.73)	35.98–44.53 (40.02)
Forebody length as % body length	31.91–37.36 (35.31)	37.2–38.57 (37.89)	33.3–39.86 (36.02)
Oral sucker length	253–414 (329)	317–453 (385)	268–454 (328)
Oral sucker width	340–447 (375)	328–466 (397)	284–492 (356)
Pharynx length	192–264 (227)	229–379 (304)	191–347 (234)
Pharynx width	191–261 (217)	204–289 (247)	164–274 (214)
Pharynx length as % body length	8.26–9.64 (8.92)	11.98–12.60 (12.29)	8.11–11.6 (9.98)
Pharynx length : oral sucker length	1:0.64–0.96 (0.70)	1:0.72–0.84 (0.78)	1:0.62–0.82 (0.71)
Oesophagus length	45–89 (76)	42–89 (66)	41–96 (65)
Ventral sucker length	422–507 (450)	430–564 (497)	350–593 (432)
Ventral sucker width	482–572 (505)	528–696 (612)	360–670 (501)
Oral sucker length : ventral sucker length	1:1.22–1.71 (1.39)	1:1.25–1.36 (1.31)	1:1.22–1.48 (1.30)
Oral sucker width : ventral sucker width	1:1.28–1.45 (1.35)	1:1.49–1.61 (1.55)	1:1.27–1.60 (1.40)
Genital pore to ventral sucker	192–282 (229)	101–240 (171)	112–290 (191)
Genital pore to ventral sucker as % body length	8.08–10.40 (9.04)	5.29–7.98 (6.63)	4.99–10.70 (8.14)
Intestinal bifurcation to ventral sucker anterior end	216–420 (298)	136–203 (170)	162–256 (213)
Intestinal bifurcation to ventral sucker anterior end as % body length	9.77–13.13 (11.37)	6.75–7.12 (6.94)	5.42–11.85 (9.62)
Ovary length	196–263 (216)	127–264 (196)	118–269 (177)
Ovary width	189–294 (227)	179–288 (234)	133–289 (202)
Egg length	80–84 (82)	72–76 (75)	54–78 (71)
Egg width	–	45.6 (n = 1)	37–42 (40)
Anterior testis length	247–310 (273)	157–202 (180)	191–334 (246)
Anterior testis width	299–448 (366)	212–289 (251)	201–458 (322)
Anterior testis length as % body length	9.69–11.91 (10.80)	6.72–8.22 (7.47)	9.19–12.81 (10.55)
Posterior testis length	281–420 (314)	185–254 (220)	194–367 (259)
Posterior testis width	237–417 (324)	275–285 (280)	200–388 (299)
Posterior testis length as % body length	11.3–13.25 (12.30)	8.44–9.68 (9.06)	9.23–14.44 (11.08)
Seminal receptacle length	127–184 (155)	162 (n = 1)	98–182 (136)
Seminal receptacle width	147–197 (164)	128 (n = 1)	91–200 (136)
Cirrus sac length	347–522 (420)	372–524 (448)	361–621 (462)
Cirrus sac width	121–147 (137)	178–233 (206)	115–163 (139)
Cirrus sac length as % body length	13.69–17.57 (16.18)	17.42–19.47 (18.44)	17.65–22.51 (19.88)
Cirrus sac posterior end from anterior margin of ventral sucker	132–197 (171)	131–176 (154)	116–379 (207)
Cirrus sac posterior end from posterior margin of ventral sucker	238–369 (290)	294–354 (324)	132–277 (217)
Post-testicular field	331–557 (448)	294–633 (464)	302–598 (413)
Post-testicular field as % body length	14.97–20.83 (17.52)	15.38–21.04 (18.21)	15.73–19.89 (17.42)
Ventral vitellarium to anterior extremity as % body length	19.69–27.43 (23.52)	26.03–28.41 (27.22)	20.35–28.59 (24.37)
Dorsal vitellarium to anterior extremity as % body length	14.31–22.66 (18.22)	19.48–25.85 (22.67)	18.29–24.17 (21.30)

Body elongate-oval, widest at level of ventral sucker or ovary. Tegument unarmed. Forebody 764–1,138 (900) long, representing 31.91–37.36% (35.31%) of total body length.

Oral sucker transversely-oval, subterminal. Prepharynx short. Pharynx muscular, elongate-oval. Multiple secretory glands bilateral to pharynx, from posterior margin of oral sucker to level of intestinal bifurcation. Oesophagus short. Intestinal bifurcation in posterior half of forebody. Caeca with wide lumen and distinctly thick epithelial lining, terminate blindly near posterior body extremity. Ventral sucker transversely-oval, larger than oral sucker, equatorial to pre-equatorial.

Testes two, postovarian, margins entire, tandem in mid-hindbody. Anterior testis transversely-oval, posterior margin overlap anterior margin of posterior testis ventrally. Posterior testis elongate-oval to subspherical. Post-testicular field occupying 14.97–20.83% (17.52%) of body length. Cirrus sac elongate, median and curving slightly dextral, overlap anterior third or mid-level of ventral sucker, rarely anterior margin of ventral sucker. Internal seminal vesicle elongate, tubular, convoluted, widens posteriorly, occupies most of cirrus sac. *Pars prostatica* and ejaculatory duct poorly defined from seminal vesicle, not characterised. Genital atrium restricted to region immediately preceding genital pore. Genital pore ventro-sinistral, at level of oesophagus or intestinal bifurcation.

Ovary subspherical to transversely-oval, margins entire, antero-dextral to anterior testis, occasionally overlap anterior end of anterior testis ventrally, may be contiguous with or slightly overlap posterior margin of ventral sucker. Mehlis’ gland rarely observed, sinistral to ovary. Canalicular seminal receptacle elongate-oval, dorsal to ovary, sometimes contiguous with or overlap anterior margin of anterior testis. Laurer’s canal not observed. Uterus restricted to region between posterior margin of anterior testis and genital pore, sinuous, filled with eggs. Metraterm distinct, long, forms distal portion of uterus, leads to genital atrium. Eggs oval, operculate, yellow, non-filamented. Vitellarium follicular; vitelline follicles arranged in numerous irregularly spheroid bundles; vitelline fields in ventral view not confluent in forebody, extend from level of intestinal bifurcation or posterior margin of pharynx to posterior body extremity, slightly overlap caeca, testes and ovary ventrally, confluent in post-testicular field; vitelline fields in dorsal view confluent in forebody but not at level of ventral sucker, extend from posterior half of pharynx or level of oesophagus to posterior body extremity, confluent in hindbody posterior to ovary, overlap caeca, testes and ovary, always extend slightly more anteriorly than follicles in ventral view. Transverse vitelline ducts medially confluent, form vitelline reservoir dorsal to ovary.

Excretory pore median, terminal. Excretory vesicle dorsal to testes, mostly obscured by vitellarium, full extent not observed past posterior testis, excretory arms reaching level of pharynx.

### ***Macvicaria tsitsikamma*** n. sp.

#### Taxonomic summary

Type-host: *Diplodus capensis*.

Type-locality: Tsitsikamma section of the Garden Route National Park, South Africa (34°1'15.2" S, 23°52'43.2" E).

Site of infection: Intestine.

Type-material: Holotype (NMB P1212) and one stained and permanently mounted paratype specimen deposited in NMB (NMB P1213).

Representative DNA sequences: two sequences of 28S rDNA (PZ068326–PZ068327), two sequences of ITS2 rDNA (PZ068337–PZ068338), two sequences of *cox*1 mtDNA (PZ067598–PZ067599), and one sequence of *nad*1 mtDNA (PZ117060).

Zoobank ID: The species *Macvicaria tsitsikamma* was registered in ZooBank under the LSID urn:lsid:zoobank.org:act:2E586BF8-1496-4B86-B7A0-0D13A5B9E70A.

Etymology: The species is named after the Khoisan word *tsitsikamma* meaning “place of many waters” or “abundance of water”, which is also reflected in the name of the type-locality, the Tsitsikamma section of the Garden Route National Park. The species name is a noun in apposition.

Description (based on 2 whole mounts; Fig. 2b, e; Table 4, S1)

Body elongate-oval, widest at level of ventral sucker. Tegument unarmed, rugose. Forebody 737–1,119 (928) long, representing 37.20–38.57% (37.89%) of body length.

Oral sucker subspherical to slightly elongate-oval, subterminal. Prepharynx short. Pharynx muscular, elongate-oval. Multiple secretory glands bilateral to pharynx, from posterior margin of oral sucker to end of pharynx. Oesophagus short. Intestinal bifurcation in posterior third of forebody. Caeca with distinct epithelial lining, terminate blindly near posterior body extremity. Ventral sucker transversely-oval, larger than oral sucker, equatorial.

Testes two, postovarian, margins entire, tandem or slightly obliquely tandem in mid-hindbody. Anterior testis transversely-oval, posterior margin overlap anterior margin of posterior testis ventrally, contiguous to or overlap ovary. Posterior testis elongate-oval. Post-testicular field 15.38–21.04% (18.21%) of body length. Cirrus sac elongate, median and curving slightly dextral, overlap anterior third of ventral sucker. Internal seminal vesicle elongate, tubular, convoluted, widens posteriorly, occupies most of cirrus sac. *Pars prostatica* and ejaculatory duct poorly defined from seminal vesicle, not characterised. Genital atrium restricted to region immediately preceding genital pore. Genital pore ventral, sinistral to median, mid-way between pharynx and ventral sucker or near posterior end of pharynx.

Ovary subspherical, margins entire, antero-dextral to anterior testis, may be contiguous with or dorsally overlap posterior margin of ventral sucker. Mehlis’ gland not observed. Canalicular seminal receptacle subspherical to elongate-oval, dorsal to ovary, contiguous with or overlap anterior margin of anterior testis. Laurer’s canal not observed. Uterus restricted to region between anterior testis and genital pore, sinuous, filled with eggs. Metraterm distinct, long, forms distal portion of uterus, leads to genital atrium. Eggs oval, operculate, yellow, non-filamented. Vitellarium follicular; vitelline follicles arranged in numerous irregularly spheroid bundles; vitelline fields in ventral view not confluent in forebody, extend from posterior half or posterior margin of pharynx to posterior body extremity, ventro-laterally overlap testes, ovary and caeca, confluent in post-testicular field; vitelline fields in dorsal view extend from posterior half or posterior margin of pharynx to posterior body extremity, confluent in forebody but not at level of ventral sucker, confluent in hindbody posterior to ovary, overlap caeca, testes and ovary, always extends slightly more anteriorly than follicles in ventral view. Vitelline reservoir dorsal to ovary.

Excretory pore median, terminal. Excretory vesicle mostly obscured by vitellarium, excretory arms reaching level of pharynx.

### *Macvicaria umbungu* n. sp.

#### Taxonomic summary

Type-host: *Diplodus capensis*.

Other hosts: Zebra seabream, *Diplodus hottentotus* (Smith) (Sparidae).

Type-locality: De Hoop Nature Reserve, South Africa (34°28'42.1" S, 20°30'39.9" E).

Other localities: Mossel Bay (34°10'45.3" S, 22°09'07" E); Tsitsikamma section of the Garden Route National Park (34°1'15.2" S, 23°52'43.2" E); Boknesstrand (33°43'54.9" S, 26°34'54.1" E); Chintsa East (32°50'11.5" S, 28°7'1.1" E), South Africa.

Site of infection: Intestine.

Type-material: Holotype (NMB P1197) and 14 stained and permanently mounted paratype specimens deposited in NMB (NMB P1198–1211); four stained and permanently mounted paratype specimens deposited in ISAM (SAMC-A100001).

Representative DNA sequences: one sequence of 18S rDNA (PZ068324), six partial sequences of 28S rDNA (PZ068328–PZ068333), two sequences of ITS1-5.8S-ITS2 rDNA (PZ068334–PZ068335), five sequences of ITS2 rDNA (PZ068339–PZ068343), 21 sequences of *cox*1 mtDNA (PZ067600–PZ067620), and 13 sequences of *nad*1 mtDNA (PZ117061–PZ117073).

Zoobank ID: The species *Macvicaria umbungu* was registered in ZooBank under the LSID urn:lsid:zoobank.org:act:CBBB5AB5-63C1-463A-A946-5B369FF54A7F.

Etymology: The species name, noun in apposition, is derived from the isiXhosa (a native South African language) word *umbungu*, meaning “worm”, in reference to the common description of digeneans as worms.

Description (based on 13 whole mounts; Fig. 2c, f; Table 4, S1)

Body elongate-oval, widest at level of ventral sucker. Tegument thick, unarmed. Forebody 671–1152 (826) long, representing 33.3–39.9% (36%) of total body length.

Oral sucker subspherical to slightly elongate-oval, subterminal. Prepharynx short. Pharynx muscular, elongate-oval. Multiple secretory glands bilateral to pharynx, from posterior margin of oral sucker to posterior quarter of pharynx. Oesophagus short. Intestinal bifurcation in posterior third of forebody. Caeca with wide lumen and distinct epithelial lining, terminate blindly near posterior body extremity. Ventral sucker transversely-oval, larger than oral sucker, slightly pre-equatorial to equatorial.

Testes two, postovarian, margins entire, tandem in mid-hindbody. Anterior testis subspherical to transversely-oval, posterior margin overlap anterior margin of posterior testis ventrally. Posterior testis subspherical to transversely-oval. Post-testicular field occupying 15.7–19.9% (17.4%) of total body length. Cirrus sac elongate, median and curving slightly dextral, overlap middle or occasionally anterior third of ventral sucker, rarely overlaps posterior third of ventral sucker. Internal seminal vesicle elongate, tubular, convoluted, widens posteriorly, occupies most of cirrus sac. *Pars prostatica* and ejaculatory duct poorly defined from seminal vesicle, not characterised. Genital atrium restricted to region immediately preceding genital pore. Genital pore ventro-sinistral, at level of intestinal bifurcation or just posterior to pharynx.

Ovary subspherical to transversely-oval, margins entire, antero-dextral to anterior testis, often contiguous with or slightly overlap anterior testis ventrally, sometimes not contiguous with anterior testis, often contiguous with or slightly overlap posterior margin of ventral sucker dorsally. Mehlis’ gland rarely observed, sinistral to ovary. Canalicular seminal receptacle subspherical to slightly elongate-oval, dorsal to ovary, contiguous with or overlap anterior margin of anterior testis. Laurer’s canal not observed. Uterus restricted to region between anterior testis and genital pore, sinuous, filled with eggs. Metraterm distinct, long, forms distal portion of uterus, leads to genital atrium. Eggs oval, operculate, yellow, non-filamented. Vitellarium follicular; vitelline follicles arranged in numerous irregularly spheroid bundles; vitelline fields in ventral view often not confluent in forebody, extend from posterior margin of pharynx or level of intestinal bifurcation to posterior body extremity, overlap caeca and slightly overlap testes, confluent in post-testicular field; vitelline fields in dorsal view extend from middle of or just posterior to pharynx to posterior body extremity, confluent in forebody but not at level of ventral sucker, confluent in hindbody posterior to ovary, mostly concentrated in lateral hind-body, overlap caeca and testes, always extends slightly more anteriorly than follicles in ventral view. Transverse vitelline ducts medially confluent, form vitelline reservoir dorsal to ovary.

Excretory vesicle mostly obscured by vitellarium, excretory arms reaching level of pharynx. Excretory pore median, terminal.

### Remarks

As is common for species of *Macvicaria*, the three new species cannot be readily distinguished based on a single diagnostic character. However, of the three new species, *M*. *tsitsikamma* exhibits several morphological characteristics that are distinct from the other two new species, including a larger pharynx, larger suckers, shorter hindbody, longer post-testicular field, distinctly smaller testes, vitelline follicles that commence furthest from the anterior extremity on average, the position of the genital pore, intestinal bifurcation being closer to the ventral sucker, and the cirrus sac extending the shortest distance beyond the anterior margin of the ventral sucker (based on average measurements as seen in Table 4). *Macvicaria peetvermaaki* can be distinguished from the two other species described in this study in having a more elongate body, the longest hindbody, the largest testes, intestinal bifurcation that is situated much further anterior to the ventral sucker, vitelline follicles that are positioned closer to the anterior extremity, shortest cirrus sac in relation to body length, and considerably larger eggs (Table 4). Additionally, *M*. *umbungu* differs from the other two new species in having (on average) a longer cirrus sac in relation to total body length, and shorter forebody; it is also the only species where the cirrus sac may extend into the posterior half of the ventral sucker (Table 4).

Aken’Ova et al. (2008) classified species of *Macvicaria* in six groups (named A–F) based on the distribution of vitelline follicles, the extent of the cirrus sac and the arrangement of testes. According to this system of classification, all species from the present study form part of Group B by possessing vitelline fields that are laterally continuous at the level of the ventral sucker, testes that are positioned in tandem, and a cirrus sac that does not extend posterior to the ventral sucker. To date, Group B consists of the 17 species as classified by Aken’Ova et al. (2008), as well as five species synonymised or described thereafter, which also conform to these morphological characteristics: *Macvicaria bartolii* Antar, Georgieva, Gargouri & Kostadinova, 2015, *Macvicaria gibsoni* Rima, Marzoug, Pérez-del-Olmo, Kostadinova, Bouderbala & Georgieva, 2017, *Macvicaria indica* (Gupta & Sehgal, 1971), *Macvicaria magellanica* Laskowski, Jeżewski & Zdzitowiecki, 2013 and *Macvicaria microlepis* (Salman & Srivastava, 1990).

The newly described species differ morphologically from the other members of Group B for which molecular data are available, as follows: from *M*. *bartolii* by having a larger body, pharynx and testes (Antar et al., 2015); from *M*. *crassigula* by having a larger body, suckers, and pharynx, additionally *M*. *tsitsikamma* and *M*. *peetvermaaki* have larger eggs (Linton, 1910); from *M*. *dubia* by having higher upper limits for body size (Stossich, 1905); from *M*. *gibsoni* by having higher upper limits for body size, a longer forebody, narrower body compared to length, larger oral sucker, and notably narrower eggs (Rima et al., 2017); from *M*. *magellanica* by having a larger body, larger pharynx, shorter oesophagus, larger suckers, and by having a seminal vesicle that is not bipartite and a cirrus sac that does not reach posterior to the ventral sucker (Laskowski et al., 2013); from *Macvicaria maillardi* Bartoli, Bray & Gibson, 1989 by having much lower upper limits for body, suckers, oesophagus, ovary, testes and cirrus sac size (Bartoli et al., 1989); from *Macvicaria mormyri* (Stossich, 1885) by having higher upper limits for body size and a cirrus sac that does not extend posterior to the ventral sucker (Stossich, 1885); and from *M*. *obovata* by having much lower upper limits for body, suckers and pharynx size, as well as a shorter oesophagus, narrower testes and longer cirrus sac (Molin, 1859).

In addition to differences in geographical distribution and host species, specimens from the present study can be distinguished morphologically from the remaining species classified in Group B, for which no molecular data are available. The three species described herein are notably larger than *Macvicaria dactylopagri* (Manter, 1954), *Macvicaria hunghuanensis* (Qiu & Li in Shen & Qiu, 1995) and *M*. *indica* in body, suckers, pharynx and egg size (Gupta & Sehgal, 1971; Manter, 1954; Shen & Qiu, 1995). *Macvicaria cynoglossi* (Madhavi, 1975) can be distinguished from the three new species by having a notably longer body, smaller oral sucker and pharynx, wider eggs, testes that are slightly lobed, and a cirrus sac that does not extend as far posterior to the anterior margin of the ventral sucker (Madhavi, 1975). *Macvicaria longicaudus* (Hafeezullah, 1971) differs from the three new species by the cirrus sac that never overlaps the ventral sucker, as well as having smaller suckers, ovary and pharynx (Hafeezullah, 1971). The three new species differ from *Macvicaria mekistomorphe* Aken’Ova, Cribb & Bray, 2008 by being larger, having a larger pharynx, suckers, ovary, cirrus sac, and testes, as well as the absence of papillate structures on the tegument and the testes being contiguous (Aken’Ova et al., 2008). *Macvicaria microlepis* can easily be distinguished from the three new species by having a bipartite seminal vesicle, a longer oesophagus, and higher upper limits for body, sucker and ovary size (Salman & Srivastava, 1990). Aside from being wider, having a larger oral sucker, larger pharynx, shorter oesophagus and narrower cirrus sac, the three new species also differ from *Macvicaria ophthalmolyci* Zdzitowiecki, 1990 by having a cirrus sac that does not reach posterior to the ventral sucker and a body that is not so tapered posteriorly (Zdzitowiecki, 1990). The three new species described herein can also be distinguished from *Macvicaria eleuthoronemae* (Wang, Wang & Zhang, 1992), *Macvicaria shotteri* Aken’Ova, Cribb & Bray, 2008, *Macvicaria sillagonis* (Yamaguti, 1938) and *Macvicaria taksengi* Bray, 1985 by having considerably higher upper limits for body size, as well as larger suckers, pharynx, and ovary (Yamaguti, 1938; Bray, 1985; Wang et al., 1992; Aken’Ova et al., 2008). *Macvicaria chrysophrys* (Nagaty & Abdel Aal, 1969) can be differentiated from the three new species by having considerably higher upper limits for body, suckers, and pharynx size, as well as having much larger testes and a smaller seminal receptacle (Nagaty & Abdel Aal, 1969).

Compared to others in this group, the three new species from southern Africa most closely resemble *Macvicaria aegyptensis* (Shalaby & Hassanine, 1997) described from *Acanthopagrus bifasciatus* (Forsskål) (Sparidae) from the Egyptian Red Sea. However, *M. aegyptensis* has considerably higher upper limits for body size, a much wider pharynx, wider ventral sucker, higher upper limits for testes size, and the eggs are considerably shorter than those of *M. tsitsikamma* and *M. peetvermaaki* (Shalaby & Hassanine, 1997).

### Molecular characterisation

DNA-based analyses of the 28S rDNA, *cox*1 mtDNA, and *nad*1 mtDNA sequences confirmed that the three species described here are distinct from each other and from all other species of *Macvicaria* for which sequence data are available.

**Fig. 3:**
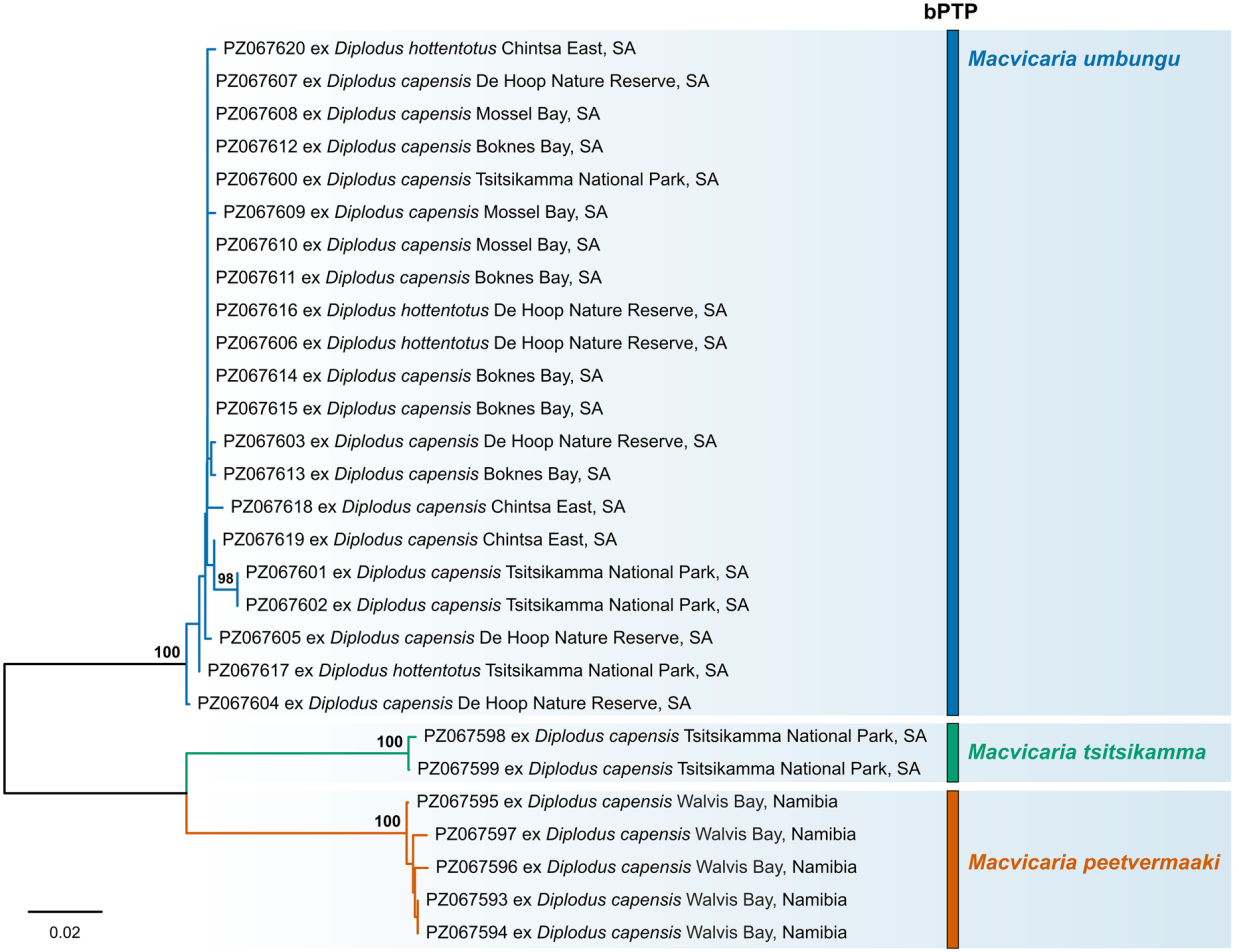
Unrooted cladogram resulting from Neighbour-Joining (NJ) analysis of the *cox*1 mtDNA sequences of *Macvicaria* spp. generated in the present study. Nodal support values below 70 are not shown. Species boundaries of novel isolates of *Macvicaria* spp. inferred with the bPTP approach are plotted on the right-hand side.

A total of 28 *cox*1 mtDNA sequences were generated for the newly collected isolates of *Macvicaria* spp. (Table 2). Neighbour-joining analysis of the *cox*1 mtDNA alignment (474 nt) produced three distinct well-supported clades, each corresponding to one of the new species of *Macvicaria* (Fig. 3). The intraspecific variation was 0–1.5% (0–7 nt) within the clade representing isolates of *M. umbungu*, 0.2% (1 nt) within the clade representing isolates of *M*. *tsitsikamma*, and 0–0.8% (0–4 nt) within the clade representing *M*. *peetvermaaki*.

The lowest interspecific divergence in the *cox*1 mtDNA region was observed between the sequences of *M*. *tsitsikamma* and *M*. *peetvermaaki*, 11.2–11.6% (53–55 nt), whereas the highest divergence was observed between *M*. *umbungu* and *M*. *peetvermaaki*, 14.6–15.8% (69–75 nt). The interspecific divergence between sequences of *M*. *umbungu* and *M*. *tsitsikamma* was 14.1–15.2% (67–72 nt). The *nad*1 mtDNA sequences were only successfully generated for isolates of *M*. *umbungu* and *M*. *tsitsikamma* (Table 2). The intraspecific divergence between *nad*1 sequences of *M*. *umbungu* was 0–0.7% (0–3 nt). The interspecific divergence between *nad*1 mtDNA sequences of *M*. *umbungu* and *M*. *tsitsikamma* was much higher than between the *cox*1 mtDNA sequences of these species, being 37.4–38.7% (135–162 nt). Due to the absence of the *cox*1 and *nad*1 mtDNA sequences for species of this genus in GenBank, the novel sequences were only used to compare sequence divergence.

**Fig. 4:**
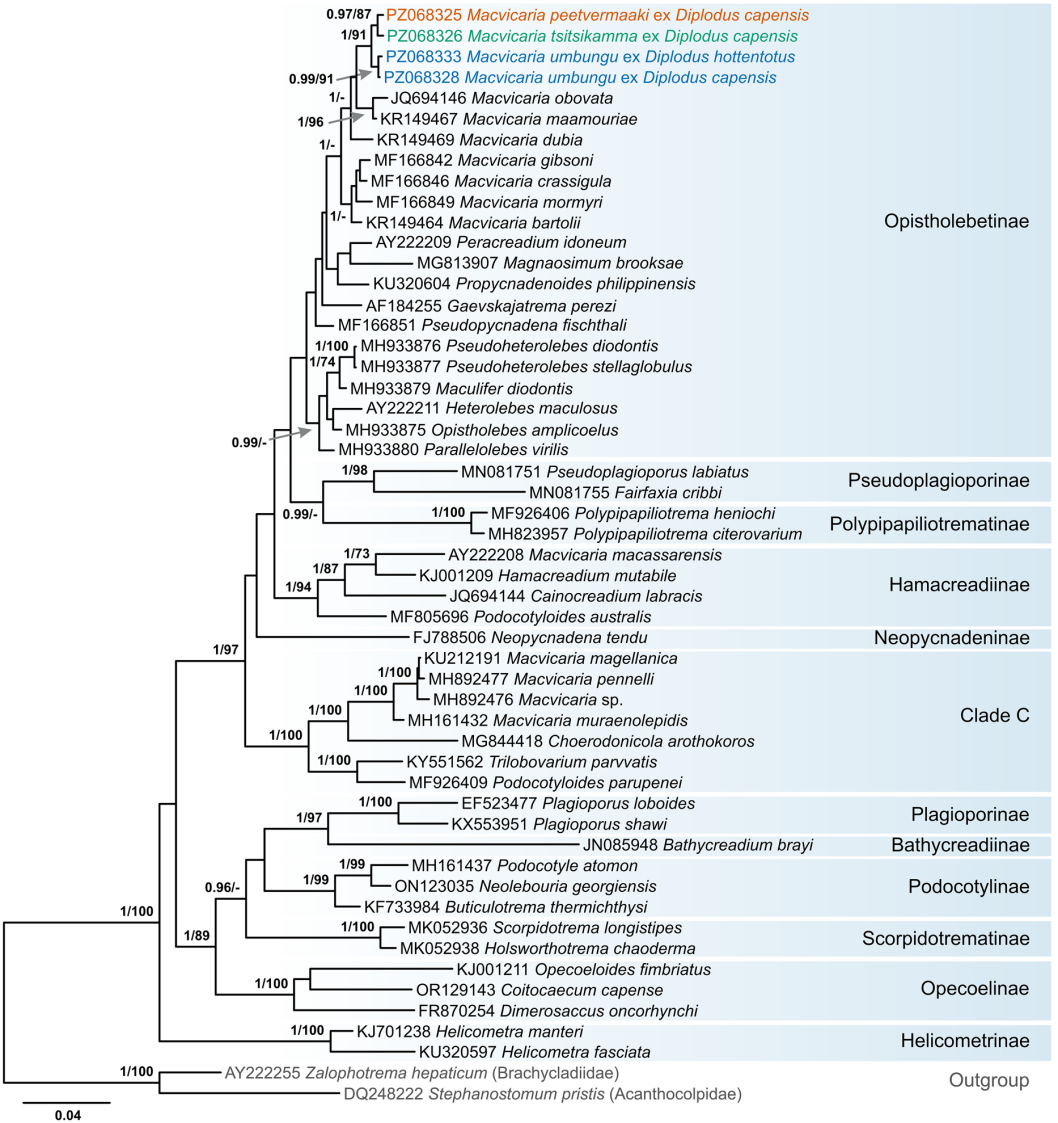
Bayesian inference (BI) phylogenetic tree of the Opecoelidae, based on the 28S rDNA dataset. Nodal support given as BI posterior probabilities/Maximum likelihood bootstraps (ML). Values below 0.9 for BI and 70 for ML are not shown. Newly sequenced isolates are highlighted with colour (blue, *Macvicaria umbungu*; green, *Macvicaria tsitsikamma*; orange, *Macvicaria peetvermaaki*). (Color figure online)

**Fig. 5:**
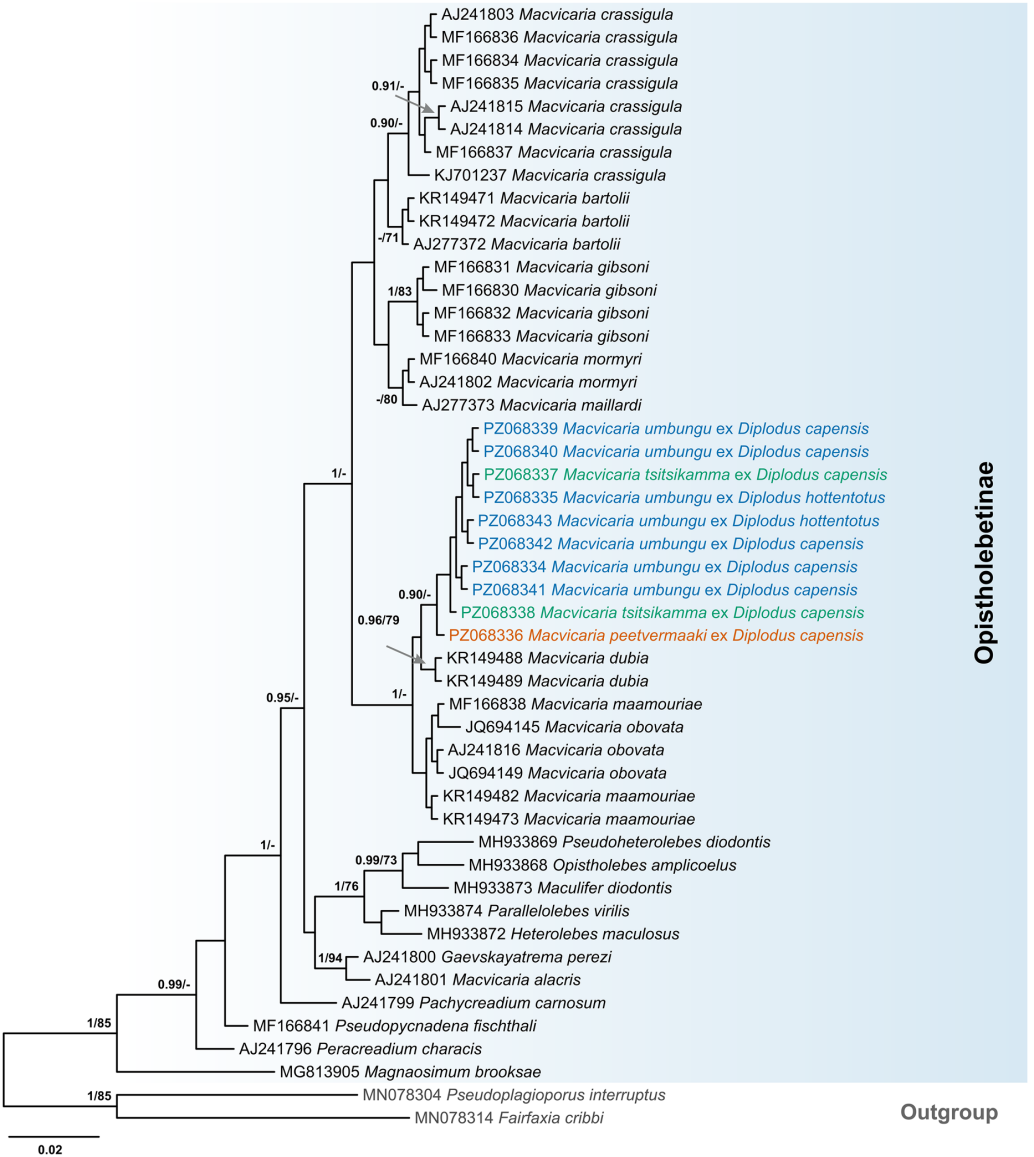
Bayesian inference (BI) phylogenetic tree of the Opecoelidae, based on the ITS2 rDNA dataset. Nodal support given as BI posterior probabilities/Maximum likelihood bootstraps (ML). Values below 0.9 for BI and 70 for ML are not shown. Newly sequenced isolates are highlighted with colour (blue, *Macvicaria umbungu*; green, *Macvicaria tsitsikamma*; orange, *Macvicaria peetvermaaki*). (Color figure online)

The sequences of the partial 28S and ITS2 rDNA regions were analysed against representatives of the Opecoelidae retrieved from GenBank (Table 3, Figs. 4, 5). In the partial 28S rDNA analyses (1,146 nt), newly sequenced isolates formed a well-supported clade among the Opistholebetinae, distinct from all other species of *Macvicaria*, except those from the Mediterranean, which formed a sister clade (*Macvicaria dubia* (Stossich, 1905),* Macvicaria maamouriae* Antar, Georgieva, Gargouri & Kostadinova, 2015 and *M. obovata*) (Fig. 4). The intraspecific variation between the partial 28S rDNA sequences of *M*. *umbungu* was 0–0.1% (0–1 nt) and the two sequences of *M*. *tsitsikamma* were identical. The interspecific divergence between the three new species of *Macvicaria* ranged between 0.3 and 0.7% (4–8 nt), with the lowest divergence being between *M*. *tsitsikamma* and *M*. *peetvermaaki* and the highest divergence between *M*. *umbungu* and *M*. *peetvermaaki*.

The three novel taxa formed a clade with other members of the Opistholebetinae in analyses of the ITS2 rDNA region, but species-level differences between our new taxa were poorly resolved, with sequences of *M*. *umbungu* and *M*. *tsitsikamma* being identical and sequences of *M*. *peetvermaaki* differing from both by 0.2% (1 nt) (Fig. 5).

**Fig. 6:**
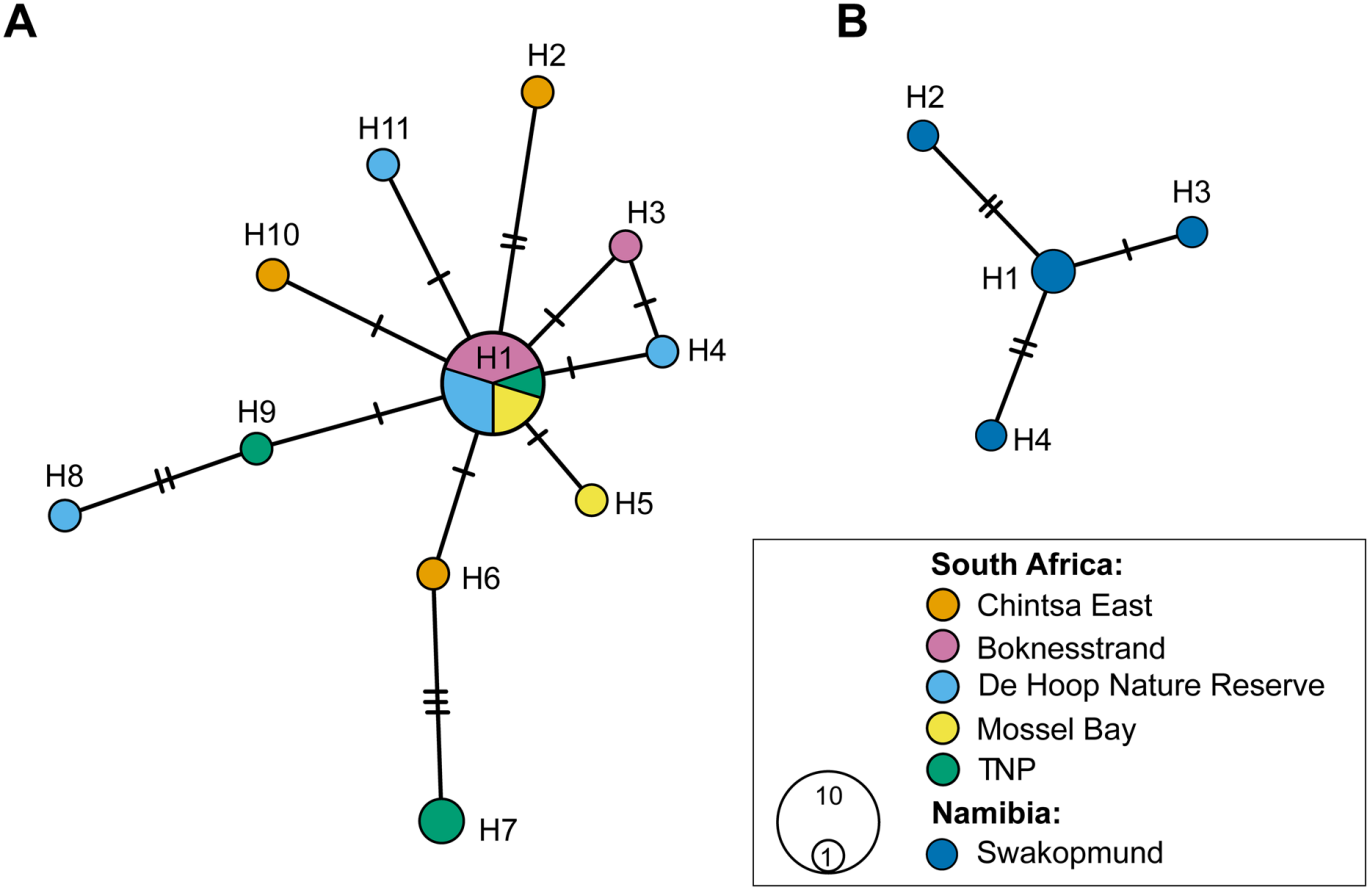
Haplotype network for *Macvicaria* spp. based on novel *cox*1 mtDNA sequences from isolates collected at six localities along the southern African coast from *Diplodus capensis* and *Diplodus hottentotus*. An unsampled intermediate haplotype is represented by a short intersecting line; each branch corresponds to a single mutational difference and connective lines represent one mutational step. Circle size is proportional to the number of isolates sharing a haplotype; haplotype frequency is indicated by colourless circles. Numbers indicate the haplotype code number (see Table 2 for details). **A** haplotype network of *Macvicaria umbungu*; ** B** haplotype network of *Macvicaria peetvermaaki*. *H* haplotype

The newly generated *cox*1 mtDNA sequences comprise 11 haplotypes for *M*. *umbungu*, two for *M*. *tsitsikamma*, and four for *M*. *peetvermaaki* (Fig. 6). Since sequences for only two isolates of *M*. *tsitsikamma* were generated, and they both represented unique haplotypes, they were not included in the figure. Out of 11 haplotypes of *M*. *umbungu* sampled from *D. capensis* and *D. hottentotus*, nine were unique and two (H1 and H7) were shared (see Table 2 for details). H7 was shared between two isolates collected from *D. capensis* in the Tsitsikamma section of the Garden Route National Park, whereas H1 was shared between 10 isolates found in four sampling sites, from the easternmost locality of our sampling range, Boknesstrand, to the westernmost, De Hoop Nature Reserve (Fig. 6), where the isolates of H1 were found in both *D*. *capensis* and *D*. *hottentotus*. Thus, the genetic variability within this species is not based on locality or host species. Out of four haplotypes of *M*. *peetvermaaki* collected in Namibia from *D. capensis,* three (H2–H4) were unique and one (H1) was shared by two isolates.

## Discussion

The genus *Macvicaria* currently comprises 58 recognised species, including our three new taxa (WoRMS, 2025); these are known from a range of taxonomically disparate fish hosts and geographical regions. In recent studies exploring the molecular phylogenetic relationships of the Opecoelidae, *Macvicaria* was demonstrated to be polyphyletic (Bray et al., 2016; Faltýnková et al., 2017; Martin et al., 2020b). *Macvicaria macassarensis* (Yamaguti, 1952), a species described from lethrinid fishes in the Tropical Indo-West Pacific (TIWP), clustered together with members of the subfamily Hamacreadiinae. The species of *Macvicaria* occurring in fishes from the Antarctic and sub-Antarctic waters mostly cluster with members of the subfamily Plagioporinae (*sensu lato*) “clade C” *sensu* Martin et al. (2018b). Species known mainly from sparid fishes in the Mediterranean, as well as the type-species *Macvicaria alacris* (Looss, 1901), clustered within the subfamily Opistholebetinae. This suggests that the species currently assigned to *Macvicaria* should be reclassified into at least three genera. The new species described in the present study resolved within the Opistholebetinae based on both the 28S and ITS2 rDNA analyses, forming a main clade with two other subclades formed by the Mediterranean species of *Macvicaria*.

Like many representatives of the Opecoelidae, species of *Macvicaria* are notoriously difficult to identify due to their high morphological similarity. However, except for the two species complexes reported in the Mediterranean (‘‘*M. obovata*’’ and ‘‘*M. crassigula*’’), all recognised species of the genus can be morphologically distinguished (Martin et al., 2018b). Following the criteria for species recognition of digenean trematodes proposed by Bray et al. (2022) and following an integrated taxonomic approach that considers multiple factors as outlined by Cribb et al. (2025), the three new species demonstrated (i) separate monophyletic groups (reciprocal monophyly) at species level based on the 28S rDNA and *cox*1 mtDNA regions; (ii) consistent morphological/morphometric differences; and (iii) in the case of *M*. *peetvermaaki*, distinct geographic distribution.

Contrary to the genetic markers (28S rDNA, *cox*1 mtDNA and *nad*1 mtDNA) that clearly distinguished our three new species and complemented our morphological differentiation, the ITS2 rDNA region did not resolve the species-level differences observed in analyses of other gene regions and thus appeared to be a poor marker to use for species delineation. Compared to the high differences observed between *cox*1 mtDNA and *nad*1 mtDNA sequences [11.2–15.8% (53–75 nt) and 37.4–38.7% (135–162 nt), respectively], the differences between the partial 28S rDNA sequences were low [0.3–0.7% (4–8 nt)] and very low [0.2% (1 nt)] or absent between the ITS2 sequences. Such a system, wherein ITS2 rDNA shows no or lower levels of divergence relative to 28S rDNA and *cox*1 mtDNA, is relatively rare (see Table 4 of Cribb et al., 2025 for a list of similar systems). Our findings therefore suggest that future studies involving delineation of *Macvicaria* species should consider applying *cox*1 mtDNA as a highly variable genetic marker, in combination with more conserved markers, i.e. 28S rDNA.

Analyses of mitochondrial haplotypes in the present study showed strong differentiation between the South African *M*. *umbungu* and the Namibian *M*. *peetvermaaki* (sufficient to be recognised as species-level differences), but no clear intraspecific population structuring by host species or geographic location for each representative species. This pattern is consistent with high gene flow among local seabream populations, mediated by high connectivity between regional locations, but discontinuously so on the west coast. This is borne out by Olivier et al. (2025), which showed the south coastal population of *D*. *capensis* to be relatively homogenous and imperfectly separated from the western population by the Benguela Upwelling System prevailing on the western coast of southern Africa. This pattern is identical to that observed for other southern African inshore fishes, e.g. galjoen (Dichistiidae: *Dichistius capensis*) (Attwood & Cowley, 2005), geelbeck croaker (Sciaenidae: *Atractoscion aequidens*) (Henriques et al., 2014), leerfish (Carangidae: *Lichia amia*) (Henriques et al., 2012) and shallow-water Cape hake (Merluccidae: *Merluccius capensis*) (Shoopala et al., 2021). The availability of broadly-overlapping intermediate hosts would further decrease ecological and selective pressure, thus limiting deep population-level divergence (for example, Vermaak et al., 2023b; Louvard et al., 2025). A lack of population-level differentiation is nevertheless not an apparent barrier to speciation, as indicated by the presence of *M. tsitsikamma* in sympatry with *M. umbungu*. *Macvicaria tsitsikamma* forms a clade with *M. peetvermaaki* in partial 28S rDNA and *cox*1 mtDNA analyses. This suggests two possible hypotheses: 1) the infection of *Diplodus* spp. by species of *Macvicaria* in southern Africa occurred in two separate events, one by the common ancestor of *M. peetvermaaki* and *M. tsitsikamma* and another by the ancestor of *M. umbungu*; or 2) the last common ancestor of all three species infected *Diplodus* spp. in southern Africa in one relatively rapid event and subsequently speciated in allopatry off Namibia. Moreover, it is possible that the common ancestor of *M. tsitsikamma* and *M. peetvermaaki* infected *D. capensis* while the ancestor of *M. umbungu* initially infected *D. hottentotus* and subsequently switched into *D. capensis*. It is equally possible that *M. umbungu* and *M. tsitsikamma* show subtle ecological differences from each other, e.g. different intermediate hosts, facilitating speciation.

Prior to this study, only one species, *M*. *obovata,* had been reported from two sparid species and one cheilodactylid species (Bray, 1987) in South Africa (as *Pachycreadium obovata*). Since then, one of these sparid species, initially identified as *D*. *cervinus* and thereafter known as *Diplodus cervinus hottentotus*, has been elevated to species level and is now recognised as *D*. *hottentotus*. Given the proximity of the sampling localities between the present study and that of Bray (1987), where *D*. *hottentotus* were collected, it is probable that some specimens identified then as *M*. *obovata* actually represent *M*. *umbungu*. *Diplodus hottentotus* shares habitats with *D*. *capensis* and has a similar diet, which increases the likelihood of infection by the same trematode species. Although the morphometric measurements for *M*. *obovata* described by Bray (1987) from South Africa overlap with those of *M*. *umbungu*, the specimens from the present study are slightly larger in almost all aspects and have slightly smaller eggs. Furthermore, Bray (1987) mentioned that the testes of *M*. *obovata* from South Africa can be slightly indented, whereas those of *M*. *umbungu* have smooth margins. *Macvicaria obovata* has been reported by multiple authors from sparid fishes from the Mediterranean, and the only records of this species from outside the Mediterranean, including the only non-sparid records, are those of Bray (1987).

It is not unheard of for trematodes from southern Africa to have close affinities with Mediterranean taxa. Bray (1984) reported the mesometrid *Elstia stossichianum* (Monticelli, 1892), another widely known Mediterranean species that infects the salema, *Sarpa salpa* (L.) (Sparidae), from off South Africa. Other trematode species, e.g. carangid-infecting bucephalids known from the Mediterranean, were also reported by the same author from South African fishes. Vermaak et al. (2023b) found that *Proctoeces maculatus* (Looss, 1901), a fellodistomid that also infects sparid fishes in the Mediterranean, was conspecific with specimens from South Africa. Vermaak (2024) reported the opistholebetine *Pseudopycnadena fischthali* Saad-Fares & Maillard, 1986 (Opecoelidae) with two of the new *Macvicaria* species off South Africa; the former species infects sparid fishes, also in the Mediterranean (e.g. D’Amico et al., 2006, Rima et al., 2017). The present study notes the co-occurrence of *P. fischthali* with two of the new *Macvicaria* species, mirroring the findings of Rima et al. (2017), who reported a similar co-occurrence of *P. fischthali* and species of *Macvicaria* in the common two-banded seabream *Diplodus vulgaris* (Geoffroy Saint-Hilaire) from off Algeria. Most recently, Yong et al. (2025) reported two blood fluke species (Aporocotylidae) first described from Mediterranean sparid fishes, infecting fishes of the same family from off South Africa. Most such records pre-date the use of molecular sequencing techniques and identifications were thus made without the ability to consider biogeographical nuances, for both the hosts and parasites. Nevertheless, we observe a growing pattern in which the South African trematode fauna has undoubted close affinities to that of the Mediterranean fauna. This pattern extends even to other parasite groups: Sakarya et al. (2019) reported the Mediterranean caligid copepod *Lepeophthirius lichiae* Barnard, 1948 from off southern Africa, and Vermaak et al. (2025) described a new polyopisthocotylan species, *Polylabris dassie* Vermaak, Bouguerche, Acosta & Smit, 2025 infecting *D*. *capensis* off South Africa, which is identical to specimens that were genetically characterised from *D*. *vulgaris* in Algeria.

Many fish species, particularly sparids, e.g., *S*. *salpa*, *Lithognathus mormyrus* (L.) and *Lithognathus lithognathus* (Cuvier), and the carangid *L*. *amia*, occur both in the Mediterranean and off southern Africa. Still other species, such as *D*. *capensis*, were considered conspecific with Mediterranean taxa [in this case, *Diplodus sargus* (L.)] until recent authors proposed their recognition as separate species (Heemstra & Heemstra, 2004). Most of the fish species shared between the Mediterranean and southern Africa are linked *via* a connected distribution along the western African coast, a region that has been severely under-sampled for parasites, and relatively few studies have been done on the population connectivity and fine-scale biogeography of host fishes. This pattern is mirrored on the eastern African coast, with almost nothing known regarding fish movements and population structure, nor their parasite richness, between southern Africa and the Red Sea. As with the Mediterranean fauna, there is some indication that the southern African fauna has affinities to that of the Red Sea, as well as the wider TIWP region. Many fish species, e.g. the sparids *Acanthopagrus berda* (Forsskål) and *Rhabdosargus sarba* (Forsskål), among others, reach their southwestern distribution limit in southern Africa and are otherwise widespread through the Red Sea and TIWP.

Bray (1984, 1986) reported some trematode species from South African fishes previously reported from the Red Sea from similar or the same host fishes. Reimer (1985) reported the same bucephalid species, *Rhipidocotyle khalili* (Nagaty, 1937), from off Mozambique as had been previously reported from the Red Sea and off India and New Caledonia (Madhavi, 1974; Ndiaye et al., 2018). Yong et al. (2023) described a species of *Siphoderina* Manter, 1934 (Cryptogonimidae) from the lutjanid *Lutjanus fulviflamma* (Forsskål) (a widespread species in the Red Sea and TIWP) and noted that it was morphologically most similar to species of *Siphoderina* that infect the same host in the Persian Gulf. Most recently, de Klerk et al. (in press) recovered specimens of an opistholebetine opecoelid from off South Africa that were identical in both ITS2 and 28S rDNA to *Opistholebes amplicoelus *Nicoll, 1915 (Opistholebetinae) from off eastern Australia, but deeply divergent (∼15%) from them in *cox*1 mtDNA. The biogeography of trematodes in southern Africa is evidently complex. Filling in the gaps in knowledge regarding both hosts and parasites along both coastlines of continental Africa through expanded sampling will be crucial in determining the relationships between southern Africa’s fish parasite fauna and those of the Mediterranean and TIWP.

Despite its interconnected nature, the southern African coastal marine fauna is also marked by a distinctly high rate of endemism. Over 14% of southern African fish species are endemic to the region (Griffiths & Robinson, 2016), including the family Sparidae, of which one-third are found along the southern African coast (Parenti, 2019). Given the tendency toward high host-specificity among trematodes, it is likely that southern African marine trematodes likewise show similarly high levels of endemism. The factors that influence trematode endemism are not often well understood, and interpretations thereof are often confounded by highly irregular or isolated collecting. Poulin et al. (2023) demonstrated that the majority of parasites are not studied again after their first description; in our experience, this is equitable in many instances to a parasite being encountered/reported just once (at its type-locality). Ours is a rare study that has systematically assessed the parasite fauna of a single fish species (*D. capensis*) from multiple locations along the southern African coast, and thus was able to demonstrate that this fish simultaneously harbours a species of trematode that extends across much of its distribution (*M. umbungu*) and a congener that is highly localised in range (*M. tsitsikamma*). The comprehensiveness of our sampling means we are reasonably confident that *M. tsitsikamma* is specific to *D. capensis* and endemic to South Africa, if not to the one locality. Many marine species are known to form distinct populations that are driven by hydrological barriers between Namibia and South Africa (Louvard et al., 2025). Thus, considering the isolated nature of *D*. *capensis* populations along the Angolan-Namibian coast, *M*. *peetvermaaki* is also likely to be endemic to the South-east Atlantic coast. Given such biogeographic dynamics, it is likely that there is a greater diversity of *Macvicaria* yet to be discovered. Thus, the three species described in this study offer just a glimpse of the full extent of the diversity of this genus in southern African marine fishes. Further exploration and research are needed to fully uncover the diversity of *Macvicaria* and other opecoelid trematodes from this biodiverse region.

## Supplementary Information

Below is the link to the electronic supplementary material.

Supplementary file1 (XLSX 24 KB)

## Data Availability

The data that support the findings of this study are available on request from the corresponding author.
